# International league of associations for rheumatology recommendations for the management of psoriatic arthritis in resource-poor settings

**DOI:** 10.1007/s10067-020-04934-7

**Published:** 2020-01-16

**Authors:** M. Elmamoun, M. Eraso, M. Anderson, A. Maharaj, L. Coates, Vinod Chandran, on behalf of ILAR-PsA recommendations group, A. Abogamal, A. O. Adebajo, A. Ajibade, O. Ayanlowo, V. Azevedo, W. Bautista-Molano, S. Carneiro, C. Goldenstein-Schainberg, F. Hernandez-Velasco, U. Ima-Edomwonyi, A. Lima, J. Medina-Rosas, G. M. Mody, T. Narang, A. G. Ortega-Loayza, R. Ranza, A. Sharma, S. Toloza, L. Vega-Espinoza, O. Vega-Hinojosa

**Affiliations:** 1 Division of Rheumatology, Department of Medicine, University of Toronto, Toronto, Canada; 2 Centre for Prognosis Studies in the Rheumatic Diseases, Krembil Research Institute, Toronto Western Hospital, University Health Network, Toronto, Canada; 3 Library and Information Services, University Health Network, Toronto, Canada; 4 Prince Mshiyeni Memorial Hospital, Durban, South Africa; 5 University of Oxford, Oxford, UK; 6 Institute of Medical Science, University of Toronto, Toronto, Canada; 7 Department of Laboratory Medicine and Pathobiology, University of Toronto, Toronto, Canada; 8 Department of Medicine, Memorial University, St. John’s, Canada; 9 Al-Azhar Faculty of Medicine Cairo, Nasr City, Egypt; 10 University of Sheffield UK, Western Bank, Sheffield, S10 2TN UK; 11 Obafemi Awolowo University Teaching Hospitals Complex, Ile-Ife, Osun state Nigeria; 12 College of Medicine, University of Lagos/Lagos University Teaching Hospital Nigeria, Ishaga Rd, Idi-Araba, Lagos, Nigeria; 13 Federal University of Parana, Curitiba, PR Brazil; 14 University Hospital Fundación Santa Fe de Bogotá and School of Medicine Universidad Militar Nueva Granada, Bogota, Colombia; 15 State University of Rio de Janeiro and Federal University of Rio de Janeiro, Av. Pedro Calmon, 550 - Cidade Universitária da Universidade Federal do Rio de Janeiro, Rio de Janeiro, RJ 21941-901 Brazil; 16 Disciplina de Reumatologia, LIM-17, Hospital das Clinicas HCFMUSP, Faculty of Medicine - University of Sao Paulo, Sao Paulo, SP Brazil; 17 Fundación Universitaria Autónoma de las Américas, Pereira, Colombia; 18 Regional University of Blumenau (FURB), Blumenau, SC Brazil; 19 Posthumous, University of Valle, University of La Sabana, Imbanaco Medical Center, Cali, Colombia; 20 University of KwaZulu-Natal, Durban, South Africa; 21 Post Graduate Institute of Medical Education & Research, Chandigarh, 160012 India; 22 Oregon Health & Science University, 3181 S.W. Sam Jackson Park Rd, Portland, USA; 23 Rheumatology Unit, Federal University of Uberlandia, Uberlândia, MG Brazil; 24 Ministry of Health, Catamarca, Catamarca Argentina; 25 Air Force Hospital, Lima, Peru; 26 Reumacenter Clinic, Puno, Peru

**Keywords:** Africa, Latin America, Psoriasis, Spondyloarthritis, Treatment

## Abstract

**Background:**

Psoriatic arthritis (PsA) is a challenging heterogeneous disease. The European League Against Rheumatism (EULAR) and the Group for Research and Assessment of Psoriasis and PsA (GRAPPA) last published their respective recommendations for the management of PsA in 2015. However, these guidelines are primarily based on studies conducted in resource replete countries and may not be applicable in countries in the Americas (except Canada and USA) and Africa. We sought to adapt the existing recommendations for these regions under the auspices of the International League of Associations for Rheumatology (ILAR).

**Process:**

The ADAPTE Collaboration (2009) process for guideline adaptation was followed to adapt the EULAR and GRAPPA PsA treatment recommendations for the Americas and Africa. The process was conducted in three recommended phases: set-up phase; adaptation phase (defining health questions, assessing source recommendations, drafting report), and finalization phase (external review, aftercare planning, and final production).

**Result:**

ILAR recommendations have been derived principally by adapting the GRAPPA recommendations, additionally, EULAR recommendations where appropriate and supplemented by expert opinion and literature from these regions. A paucity of data relevant to resource-poor settings was found in PsA management literature.

**Conclusion:**

The ILAR Treatment Recommendations for PsA intends to serve as reference for the management of PsA in the Americas and Africa. This paper illustrates the experience of an international working group in adapting existing recommendations to a resource-poor setting. It highlights the need to conduct research on the management of PsA in these regions as data are currently lacking.

**Table:** 

**Key Points** • *The paper presents adapted recommendations for the management of psoriatic arthritis in resource-poor settings*. • *The ADAPTE process was used to adapt existing GRAPPA and EULAR recommendations by collaboration with practicing clinicians from the Americas and Africa*. • *The evidence from resource-poor settings to answer clinically relevant questions was scant or non-existent; hence, a research agenda is proposed*.

**Electronic supplementary material:**

The online version of this article (10.1007/s10067-020-04934-7) contains supplementary material, which is available to authorized users.

## Introduction

Psoriatic arthritis (PsA) is a spondyloarthritis that affects up to a third of patients with psoriasis, a common inflammatory skin disease affecting 1–3% of the population [2]. The heterogeneous disease manifestations make management of PsA a challenge [3]. The Group for Research and Assessment of Psoriasis and Psoriatic Arthritis (GRAPPA) and the European League Against Rheumatism (EULAR) have updated their respective recommendations [4, 5] for the management of PsA. These recommendations are based on systematic reviews of literature and provide evidence-based recommendations for the management of PsA. However, they are primarily based on studies conducted in resource replete countries of Europe and North America; therefore, they may not be applicable to PsA patients in resource-poor countries in the Americas excluding Canada and the USA- (henceforth termed ‘the Americas’) and Africa. To address this gap, our objective was to adapt the published GRAPPA and EULAR recommendations for the management of PsA to resource-poor settings using the ADAPTE process [6].

## Methods and results

Under the auspices of the International League of Associations for Rheumatology (ILAR), we aimed to create recommendations for the management of PsA in resource-poor settings. The recommendations were targeted at clinicians caring for PsA patients more than 16 years of age residing in the Americas or Africa. The target audience for these recommendations includes rheumatologists, dermatologists, internists, primary care practitioners, patients and other stakeholders practicing or living in the Americas or Africa. The Asia-Pacific region was not included since the Asia Pacific League of Associations for Rheumatology (APLAR) is also developing similar recommendations.

### ADAPTE process

#### Assembly of the organizing committee

An organizing committee of 8 rheumatologists with experience in PsA treatment recommendations and/or practice in resource-poor settings was established. The committee consisted of rheumatology experts, researchers, and active GRAPPA members. The committee decided to use the ADAPTE process to develop the new recommendations. The ADAPTE Collaboration [1] defines guideline adaptation as the systematic approach to considering the use and/or modification of (a) guideline(s) produced in one cultural and organizational setting for application in a different context. The process includes three phases: set-up phase, adaptation phase, and finalization phase.

### Phase one: set-up

#### Panel of participants

One hundred and thirty-four potential participants (rheumatologists and dermatologist, GRAPPA and some non-GRAPPA members of the Panamerican League of Associations for Rheumatology (PANLAR), the African League Against Rheumatism (AFLAR), and Asia-Pacific League of Associations for Rheumatology (APLAR) regions) were invited by the organizing committee to participate in an initial email survey. Members from the APLAR region were invited to provide input since they had experience in treating PsA in similar resource-poor settings. The objectives were to identify specific challenges in their local practice particularly access to specialists, access to therapies, infectious diseases, and any specific comorbidities that may influence management of PsA.

Seventy-nine respondents (57 rheumatologists and 22 dermatologists) completed the survey, of whom 16 were from Africa and 46 were from the Americas. Respondents were invited to be members of the recommendation panel. Thirty-six participants provided an affirmative response, but only 15 participants completed the project (10 rheumatologists and 5 dermatologists). The entire task force of this project represented five countries in the Americas, four countries in Africa, and four countries from other regions.

Based on the responses and a face-to-face meeting held at the annual GRAPPA meeting in 2017, the committee and the panel members identified three areas of interest to be included in the adapted recommendations: (a) efficacy and safety of pharmacotherapy, (b) recommendations for physicians with limited access to other specialists, (c) screening and management of tuberculosis (TB), hepatitis B/C virus infection (HB/CV), human immunodeficiency virus (HIV) disease, Chagas’ disease, leishmaniasis, and leprosy. Subsequently, members selected their area(s) of interest to work on; thus, three working groups were formed.

### Phase two: adaptation

#### Determining the health questions

After having identified the areas of interest, members of the committee drafted the PIPOH criteria and the health questions which was used as a tool (Table 1):

- P Patient population (including disease characteristics)
- I Intervention of interest
- P Professionals/patients (audience for whom the guideline is prepared)
- O Outcomes to be taken into consideration (purpose of the guideline)
- H Healthcare setting and context

**Table 1:** PIPOH criteria for developing the health questions

Population	PsA patients of at least 16 years of age living in the Americas or Africa, particularly those with specific comorbidities of interest(TB, HIV, HB/CV, Chagas’ disease, leishmaniasis and leprosy)
Intervention	-Screening for: TB, HIV, HB/CV, Chagas’ disease, leishmaniasis, and leprosy prior to pharmacotherapy -Adverse events during pharmacotherapy -Treatment: duration and type: sequential/combination, according to domains -Response evaluation -Supportive care-Follow up
Professionals/patient	Rheumatologists, dermatologists, internists, primary care physicians, other stakeholders, patients
Outcome	-Patient Outcomes: Drug Efficacy, adverse events -Access to specialists and multidisciplinary care
Health care setting	Hospitals, clinics, doctor’s offices, primary care.

*HIV* human immunodeficiency virus, *HB/CV* hepatitis B or C virus, *PsA* psoriatic arthritis, *TB* tuberculosis

The drafted PIPOH criteria and the health questions were disseminated via email to the entire task force for refinement. Three Patient Research Partners from the Americas also participated in this task. The PIPOH criteria and 18 questions developed are shown in Tables 1 and 2, respectively.

**Table 2:** Health questions (those marked with an asterisk* did not have sufficient evidence within the source recommendations and were included in the SLR)

Efficacy/adverse events of drug treatment	
1. What are the goals of therapy?	
2. Assessments (history, physical, laboratory and radiological) of patients, including the presence of extra articular manifestations, to achieve goals of therapy	
3. Efficacy of pharmacotherapy in all PsA domains and in the presence of extra articular manifestations	
4. Safety of pharmacotherapy in PsA	
5. Efficacy of combination therapy	
6. Safety of combination therapy*	
7. Frequency of laboratory monitoring*	
8. Safety and efficacy of biosimilars and intended copies*	
Recommendations for Rheumatologists with limited access to Dermatologists and vice versa*	
1. Recommendations to rheumatologist/internists for treatment of psoriasis particularly those with limited access to support from dermatologists	
2. Recommendations to dermatologists for treatment of psoriatic arthritis particularly those with limited access to support from rheumatologists?	
3. Recommendations for combined multidisciplinary team	
4. Availability of allied health and social support: social work, physiotherapy, occupational therapy	
TB, HB/CV, HIV, and other infections	
1. Screening for TB prior to therapy with bDMARDs*	
2. Recommendations for the management of the increased risk of TB with bDMARDs in high TB endemic areas*	
3. Recommendations on the management of infection with TB, HIV, and HB/CV in patients receiving bDMARDs*	
4. Safety of combination of bDMARDs and csDMARDs (higher risk of TB, HIV, HB/CV, Chagas’ disease, leishmaniasis, leprosy)*	
5. Screening and management of HB/CV, HIV, Chagas’ disease, leishmaniasis, leprosy*	
Assessing comorbidities and CV risk	
1. Considerations for treatment of patients with psoriatic arthritis and concomitant comorbidities*	

*bDMARDs* biological DMARD, *csDMARDs* conventional synthetic DMARDs, such as methotrexate, sulfasalazine, or leflunomide; *DMARDs* disease-modifying anti-rheumatic drugs, *HIV* human immunodeficiency virus, *HB/CV* hepatitis B/C virus, *PsA* psoriatic arthritis, *TB* tuberculosis

#### Screening source recommendations

The source recommendations were assessed on their clinical content according to the health questions formulated. We modified the ADAPTE tool 8: Table for Summarizing Guideline Content to prepare a table in which participants of each working group were asked whether an answer was stated in the source recommendations and their degree of agreement with that answer if available. After an iterative process, ten questions reached < 70% of agreement. To answer these questions, a systematic review of literature from the Americas and Africa was conducted.

#### Search for other documents: systematic literature review

The systematic search included the following databases: Medline, Embase, African Index Medicus (AIM), Cochrane Central, and Literatura Latino Americana en Ciencias de la Salud (Latin-American Literature in Health Science- LILACS); and literature identified by the panel of participants. Inclusion criteria were: (1) Randomized controlled trials, (2) observational studies, (3) case series, (4) resource-poor settings in the Americas or Africa, and (5) any language. Exclusion criteria were: (1) review articles, (2) abstracts, (3) conference proceedings, (4) case report.

**Fig. 1:**
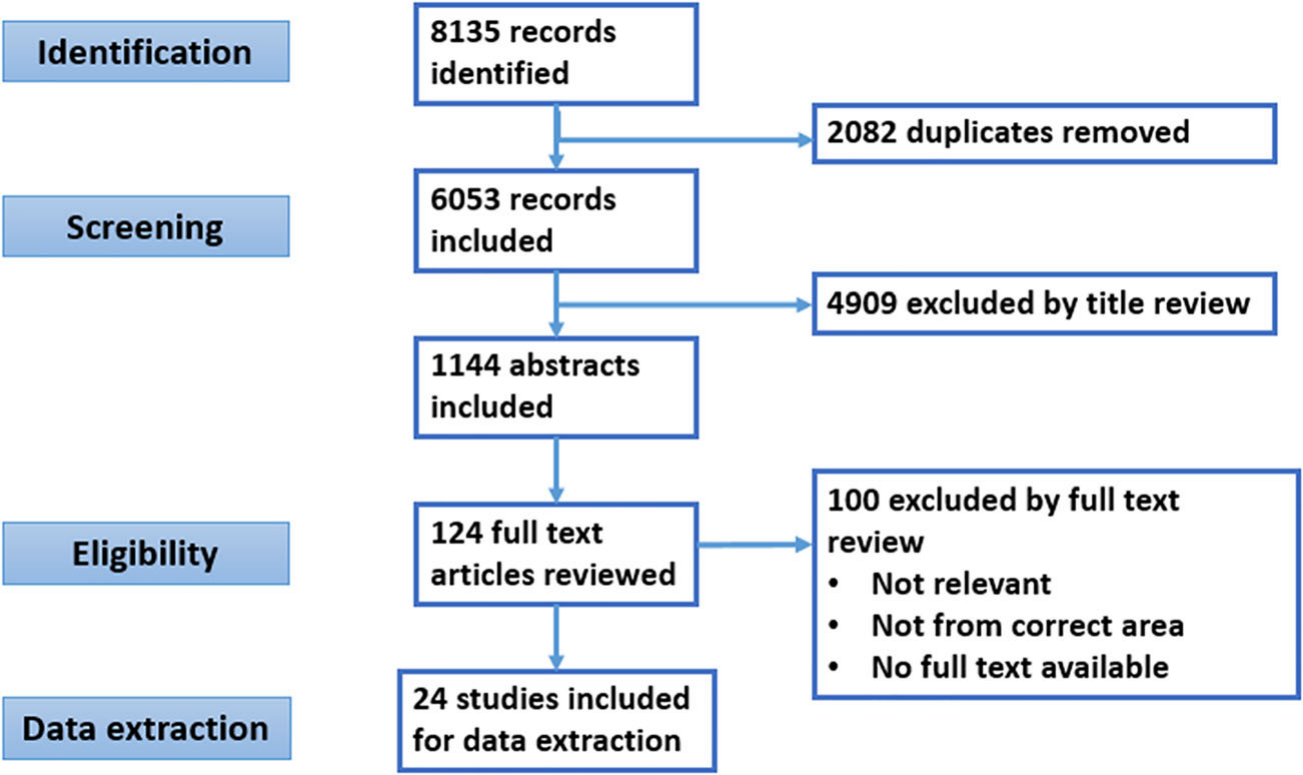
Preferred Reporting Items for Systematic Reviews and Meta-Analyses (PRISMA) flow diagram, record identification, screening, eligibility, and inclusion. The search terms included PsA, the Americas, Africa, and infectious diseases. Studies included from database inception until February 22 2018 well as literature sent by the panel. The search strategy and MESH terms are provided in Appendix 1

A systematic review to update the source recommendations was not performed since it would have been outside the scope of our objective. After duplicates were removed, articles were selected through a screening process based first on the title, second on abstract and third on the full-text review (Fig. 1). Articles were retrieved if their content was relevant to the health questions framed by the PIPOH definition for this project. Three authors carried out the data extraction independently.

The search identified 8135 articles. Of these, 24 were identified for full review and data extraction (Fig. 1). Despite this exhaustive systematic literature review (SLR), there were several health questions for this project that were not addressed by evidence retrieved. These included questions related to the safety of combinations of conventional synthetic disease-modifying anti-rheumatic drugs (csDMARDs) and biologic disease-modifying anti-rheumatic drugs (bDMARDs) in general and in areas with endemic infections, the frequency of monitoring of individuals on therapy in resource-poor settings and recommendations for dermatologists treating PsA without rheumatology support or vice versa.

Given the availability and use of biosimilar and intended copies, our search also included studies on the use of this group of drugs in PsA. One review article addressed this topic but unfortunately did not offer a clear conclusion due to a lack of evidence. When investigating the safety screening required for csDMARD or bDMARD therapy in PsA, nine studies were identified that reported screening for infectious diseases. None of these studies were reported exclusively in PsA patients and none were RCTs. The majority looked into TB screening (*n* = 8) and showed that tuberculin skin test and chest radiographs are widely used as screening tests, but the best method is still debated particularly in endemic areas.

Concerning the use of bDMARDs, 14 studies were identified that examined bDMARD use on patients in the Americas or Africa. These studies identified successful use of bDMARDs in areas of endemic infection, but limited data included meant that no recommendations different from the current ones could be made. Studies around treatment of comorbidities in PsA did not identify specific literature from the Americas or Africa.

#### Assessment of guideline quality

**Fig. 2:**
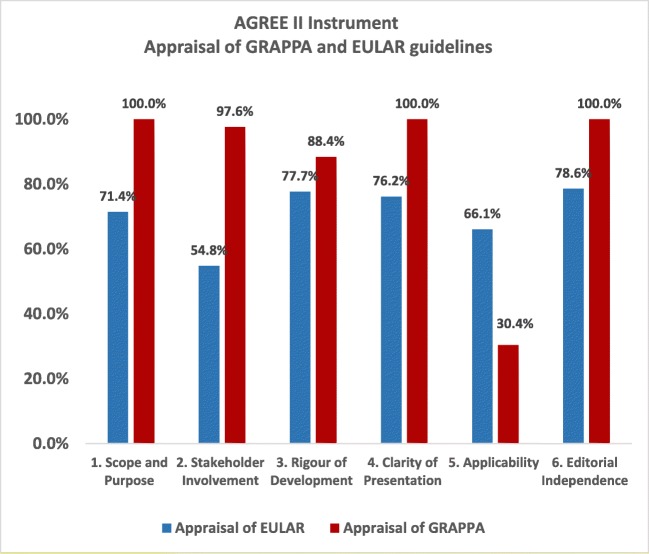
AGREE II Instrument: Appraisal of Guidelines for Research Evaluation II. Each guideline was reviewed by two independent appraisers

The quality of the GRAPPA and EULAR source recommendations was evaluated with the Appraisal of Guidelines for Research and Evaluation II (AGREE II) Instrument [7] (available at http://www.agreetrust.org/). AGREE II Instrument evaluates the process of practice guideline development and the quality of reporting by using the AGREE Reporting Checklist [8]. This instrument includes 23 items that are organized into six domains: 1. scope and purpose; 2. stakeholder involvement; 3. rigor of development; 4. clarity of presentation; 5. applicability; and 6. editorial independence. Each of the 23 items targets various aspects of practice guideline quality. Each item is scored on a scale ranging from 1 (“strongly disagree”) to 7 (“strongly agree”). Each source recommendation was independently assessed by two reviewers who upon the completion of those 23 items also provided 2 additional overall assessments of the guideline: the overall quality of the recommendation scored again from 1 to 7 and a recommendation about its use by selecting the ‘Yes’ or ‘Yes with modifications’ or ‘No’ options provided. We used the raw AGREE scores to determine agreement amongst the appraisers on various items of the AGREE domains (Fig. 2).

**Fig. 3:**
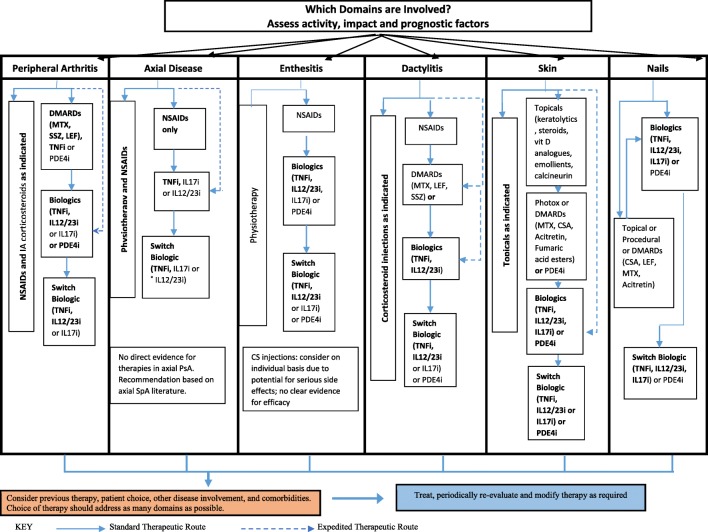
GRAPPA treatment schema, recommendations for each domain. ©2016, American College of Rheumatology. With permission from John Wiley and Sons. GRA PPA treatment schema for active psoriatic arthritis (PsA). Light text identifies conditional recommendations for drugs that do not currently have regulatory approvals or for which recommendations are based on abstract data only. CS corticosteroid, vit vitamin, CSA cyclosporine A, DMARDs disease-modifying antirheumatic drugs, IA intraarticular, IL-12/23i interleukin-12/23 inhibitor, LEF leflunomide, MTX methotrexate, NSAIDs nonsteroidal anti-inflammatory drugs, PDE-4i phosphodiesterase 4 inhibitor (apremilast), phototx phototherapy, SpA spondyloarthritis, SSZ sulfasalazine, TNFi tumor necrosis factor inhibitor

#### Assess applicability

The applicability of the principles contained in the source recommendations was assessed using Tool 15-Evaluation Sheet-Acceptability/Applicability. According to ADAPTE’s definition of acceptability and applicability, “Acceptable” indicates that it should be put it into practice, and ‘Applicable’ indicates that physicians are able to put it into practice. A table was sent to the committee members to assess the acceptability of each principle in terms of our target population, benefits to this population, and its compatibility with the culture and values of the population, and to assess the applicability of each principle in terms of availability of the intervention, expertise, legal and resource constraints. They were provided with three options: Accept as is, modify, or reject principle for further discussion (for an example see Appendix 2).

Principles from both source guidelines with a score of more than 80% in the “accept as is” option of the acceptability and applicability items were taken as overarching principles for the adapted ILAR recommendations.

#### Adaptation of the principles and the recommendations

GRAPPA and EULAR PsA treatment recommendations are recent guidelines with strong methodological quality from where principles and recommendations were adapted to produce ILAR PsA treatment recommendations for resource-poor countries. Members of the organizing committee summarized principles and recommendations from the source recommendations, and the supporting evidence of the SLR to address each health question and their applicability to the context of use according to the assessments previously described.

### Principles

The principles are shown in Table 3. Those were selected according to their acceptability and applicability with an agreement of more than 80%.

**Table 3:** Principles

A. Goals of therapy The ultimate goals of therapy for all patients with PsA are as follows: 1) To achieve the lowest possible level of disease activity in all domains of disease; as definitions of remission and low or minimal disease activity become accepted, these will be included in the goal. 2) To optimize functional status, improve quality of life and well-being, and prevent structural damage to the greatest extent possible. 3) To avoid or minimize complications, both from untreated active disease and from therapy (GRAPPA principle 1).	
B. Assessment of domains Assessment of patients with PsA requires consideration of all major disease domains, including peripheral arthritis, axial disease, enthesitis, dactylitis, psoriasis, and nail disease. The impact of disease on pain, function, quality of life, and structural damage should be examined. In addition, activity in other potential related conditions should be considered, included cardiovascular disease, uveitis and, bowel disease. Multidisciplinary and multispecialty assessment and management will be most beneficial for individual patients. (GRAPPA principle 2) PsA is a heterogeneous and potentially severe disease, which may require multidisciplinary treatment (EULAR principle A)	
C. Assessment of relevant comorbidities A comprehensive assessment of relevant comorbidities (including but not restricted to obesity, metabolic syndrome, gout, diabetes, cardiovascular disease, liver disease, depression, and anxiety) should be undertaken and documented. (GRAPPA principle 4) When managing patients with PsA, extra-articular manifestations, metabolic syndrome, cardiovascular disease and other comorbidities should be taken into account. (EULAR principle E)	
D. Safety of pharmacotherapy and shared decision making Therapeutic decisions need to be individualized, and are made jointly by the patient and his or her doctor. Treatment should reflect patient preferences, with the patients provided with the best information and relevant options provided to them. Treatment choices may be affected by various factors, including disease activity, structural damage, comorbid conditions, and previous therapies. (GRAPPA principle 5) Treatment of patients with PsA should aim at the best care and must be based on a shared decision between the patient and the rheumatologist, considering efficacy, safety and costs. (EULAR principle B)	
E. Frequency of follow-up Ideally, patients should be reviewed promptly, offered regular evaluation by appropriate specialists, and have treatment adjusted as needed in order to achieve the goals of therapy. Early diagnosis and treatment is likely to be of benefit.(GRAPPA principle 6)	

### Recommendations

Recommendations are shown in Table 4. Those were selected if their clinical content provided answers to the health questions formulated and if they reached a consensus of more than 70%. Where available, data from the literature search was used for unanswered health questions. However due to a lack of data, expert opinion was sought for the answers not found in the SLR. Although specific data were not found relating to treatment in the presence of comorbidities, this was felt to be important in all healthcare settings. Thus, the comorbidities table highlighting potential risks and benefits to comorbidities with different therapies taken from the GRAPPA recommendations was included (Table 5).

**Table 4:** Recommendations

1. Goals of therapy 1. Treatment should be aimed at reaching the target of remission or, alternatively, minimal/low disease activity, by regular monitoring and appropriate adjustment of therapy. In PsA, there exist few data regarding natural history, treatment objectives and remission. However, since in PsA inflammation is related to long-term outcomes of joint involvement, this recommendation states that the objective in patients with PsA is remission or if remission cannot be achieved, a low or minimal disease activity state. Remission is defined here as the absence of clinical and laboratory evidence of significant inflammatory disease activity. In addition to absence of inflammation in the joints, absence of enthesitis and dactylitis are also important. It should be noted that this remission of inflammation may not equate to complete absence of all symptoms for many patients. Indeed, recent work in PsA demonstrated that the impact of the disease on quality of life is related to pain, skin problems and functional disability, and fatigue, as well as emotional and social aspects of impact. Some of these aspects of impact may be less accessible to pharmacological therapies of PsA, thus leading to a ‘residual’ impact in the absence of inflammation. Furthermore, remission may be difficult to achieve in PsA. Factors associated with higher remission rates appear to be younger age, lower functional impairment and higher C reactive protein levels in some cases. Remission is still insufficiently defined in PsA. We suggest that the use of outcomes where remission/low disease activity have been defined, should be considered. This is now the case for several scores used in PsA, some of which focus only on arthritis whereas others encompass various aspects of psoriatic disease. As regards joint involvement, a stringent remission definition and criteria for low disease activity by the Disease Activity index for Psoriatic Arthritis (DAPSA) have been recently defined and validated. However, minimal/low disease may also be a relevant target especially for long-standing disease, as stringent remission may not be achievable in these patients or in some patients with comorbidities that preclude escalation of therapy. Minimal disease activity in PsA has been defined as five of the seven criteria comprising musculoskeletal and skin manifestations and patient-reported outcomes. This outcome has been shown in one study to be predictive of less structural degradation, and in the recent Tight control in PsA (TICOPA) trial to be a valid treatment target. Definitions of remission and acceptable residual disease activity levels in PsA, its predictors and its relationship with long-term outcomes are still a part of the research agenda and more thorough assessment of prognostic markers of severity (related to risk of progressive disease, structural damage, physical disability and quality of life) must still be addressed. (EULAR recommendation 1)	
2. Screening and management of TB, HIV, HB/CV, Chagas’ disease, leishmaniasis, leprosy, and other concomitant comorbidities GRAPPA treatment of PsA and concomitant comorbidities. See Table 5. Since the SLR did not find evidence to make recommendations, expert opinion is provided. Given the endemic nature of TB, HIV, HBV/HCV, Chagas’ disease, leishmaniasis, leprosy, and other infectious diseases, it is recommended that appropriate screening for prevalent infections be conducted as per local and national guidelines prior to initiation of immunosuppressive therapies (especially bDMARDs). Periodically thereafter and ideally at each clinical encounter careful assessment for active infection should be conducted to avoid serious, life threatening infectious complications.	
3. Frequency of monitoring Since the SLR did not find evidence to make recommendations, expert opinion is provided. Patients need to be evaluated periodically to assess response to therapy and identify complications and adverse events. The frequency of monitoring should depend on the time to expected response when starting a new csDMARD/bDMARD and the degree of disease activity. Less frequent follow up may be acceptable when disease is well controlled, and changes in therapy are not anticipated.	
4. Safety and efficacy of pharmacotherapy in all domains GRAPPA treatment schema, recommendations for each domain. See Fig. 3. Since the SLR did not find evidence to make recommendations about safety of bDMARDs in TB endemic areas, expert opinion is provided. We recommend that the GRAPPA recommendations on treatment be followed although we recognize that access to many therapies, especially bDMARDs may be difficult in resource poor settings. Appropriate screening for endemic disease such as tuberculosis (such as chest X-ray and Mantoux or IGRAs) prior to therapy and periodic evaluation during therapy with bDMARDs, especially TNFi agents as per local guidelines are recommended.	
5. Efficacy and safety of combination therapy Recent data suggest that continuation of a concomitant csDMARD therapy in combination with TNFis is beneficial in PsA in terms of treatment maintenance and levels of response, especially in patients using monoclonal antibodies, but more data are warranted including the effect of concomitant csDMARD on immunogenicity. (EULAR recommendation 5) Since the SLR did not find evidence to make recommendations, expert opinion is provided. The efficacy and safety of combination of biologic therapy with csDMARDs as well as combination with tsDMARD therapy is not well established. Such therapy may be used carefully with frequent monitoring of response and adverse events, especially organ toxicity and infections.	
6. Safety and efficacy of biosimilars and intended copies Since the SLR did not find evidence to make recommendations, expert opinion is provided. Use of biosimilars may be considered in the management of psoriatic arthritis with careful monitoring of adverse events, especially infections, as recommended when using bDMARDs. We do not recommend the use of intended copies, until proper evaluation of their efficacy and safety.	

*bDMARDs* biological DMARD, *csDMARDs* conventional synthetic DMARDs, such as methotrexate, sulfasalazine, or leflunomide; *DMARDs* disease-modifying anti-rheumatic drugs, *tsDMARDs* targeted synthetic DMARDs, *HIV* human immunodeficiency virus, *HB/CV* hepatitis B/C virus, *PsA* psoriatic arthritis, *SLR* systematic literature review, *TB* tuberculosis, *TNFi* tumor necrosis factor inhibitors, *GRAPPA* Group for Research and Assessment of Psoriasis and Psoriatic Arthritis, *EULAR* European League Against Rheumatism

**Table 5:** GRAPPA treatment of PsA and concomitant comorbidities. © 2016, American College of Rheumatology. With permission from John Wiley and Sons

	NSAIDs	CS	HCQ	SSZ	MTX	LEF	CSA	Etanercept	Adalimumab	Infliximab	CZP	Golimumab	Ustekinumab	Apremilast
Cardiovascular disease	C	?	NI	NI	NI	NI	NI	NI	NI	NI	NI	NI	NI	NI
Congestive heart failure	C	C	NI	NI	NI	NI	NI	C	C	C	C	C	NI	NI
Obesity	NI	NI	NI	NI	C	NI	NI	NI	NI	NI	NI	NI	NI	NI
Metabolic syndrome	NI	C	NI	NI	C	NI	NI	NI	NI	NI	NI	NI	NI	NI
Diabetes	NI	C	NI	NI	C	NI	NI	NI	NI	NI	NI	NI	NI	NI
Ulcerative colitis	?	NI	NI	A	NI	NI	OL	NI	A	A	NI	A	NI	NI
Crohn’s disease	?	NI	NI	A	OL	NI	NI	NI	A	A	A	NI	NI	NI
Uveitis	NI	P†	NI	NI	NI	NI	NI	?	P	P	NI	NI	NI	NI
Osteoporosis	NI	C	NI	NI	NI	NI	NI	NI	NI	NI	NI	NI	NI	NI
Malignancy	NI	NI	NI	NI	NI	NI	NI	C	C	C	C	C	?	NI
Fatty Liver disease	C	NI	NI	C	C	C	NI	NI	NI	NI	NI	NI	NI	NI
Chronic kidney disease	C	NI	NI	NI	C	?	SM	NI	NI	NI	NI	NI	NI	NI
Depression	NI	NI	NI	NI	NI	NI	NI	NI	NI	NI	NI	NI	NI	?
Chronic hepatitis B Ŧ	C	NI	NI	NI	C	C	NI	SM	SM	SM	SM	SM	?	NI
Chronic hepatitis C Ŧ	C	NI	NI	NI	C	C	NI	?/P	?	?	?	?	?	NI
HIV	NI	NI	NI	NI	NI	NI	NI	SM	SM	SM	SM	SM	?	NI

*A* approved for primary therapy of the comorbid condition, *C* reason for caution, *CS* corticosteroids, *CSA* cyclosporine A, *CZP* certolizumab pegol, *HCQ* hydroxychloroquine, *LEF* leflunomide, *NI* no information available, *OL* off-label use for therapy of the comorbid condition, *P* preferred therapy, *SM* requires special monitoring, *SSZ* sulfasalazine

? data insufficient but concerns have been raised.

† Corticosteroids used as preferred therapy for uveitis are most commonly given as topical and/or intraocular injections in preference to oral steroids.

Ŧ When treating patients with chronic infections that can affect the liver, consider consultation with providers who have expertise in the area.

### Phase three: finalization

#### External review and acknowledgment

After the recommendations were adapted the document was sent for external review to a dermatologist from the Americas and a rheumatologist from Africa for review. Feedback was solicited using the ADAPTE feedback questionnaire and free text. Overall the recommendations were supported and found to be beneficial. Given the inclusion of targeted therapies in the management of PsA, one reviewer felt that the recommendations were too expensive to apply given poor access to these drugs in some settings. They highlighted a relative lack of data to support the process and suggested a research agenda to be included within this recommendation (Table 6).

**Table 6:** Research Agenda for PsA in resource-poor countries to fill the gaps in the ILAR recommendations

1. Goals of therapy	• Outcomes of treatment of PsA by specialists compared to general practitioners
2. Screening and management of TB, HIV, HB/CV, Chagas’ disease, leishmaniasis, leprosy, and other concomitant comorbidities	• Cost effective screening strategy for TB, HIV, HB/CV, Chagas’ disease, leishmaniasis and leprosy • Prevalence of TB, HIV, HB/CV, Chagas’ disease, leishmaniasis, leprosy in patients with PsA • Risk of worsening or new onset TB, HIV, HB/CV, Chagas’ disease, leishmaniasis, leprosy on treatment with csDMARDs or bDMARDs in PsA • Risk of worsening or new onset TB, HIV, HB/CV, Chagas’ disease, leishmaniasis, leprosy on treatment with bDMARDs in PsA • Effect of cardiovascular and related comorbidities on achieving treatment outcome in PsA
3. Frequency of monitoring in resource poor countries	• Cost effective frequency of disease assessment/monitoring in PsA
4. Safety and efficacy of pharmacotherapy in all domains	• Safety and efficacy of csDMARDs in resource-poor settings • Safety and efficacy of bDMARDs in resource-poor settings
5. Safety and efficacy of combination therapy	• Safety and efficacy of combination csDMARDs or bDMARDs in resource-poor settings
6. Safety and efficacy of biosimilars and intended copies	• Safety and efficacy of biosimilars in resource-poor settings

*bDMARDs* biological DMARDs, *DMARD* disease-modifying anti-rheumatic drug, *HIV* human immunodeficiency virus. *HB/CV* hepatitis B/C virus, *PsA* psoriatic arthritis, *TB* tuberculosis

### Approval by endorsing bodies

These recommendations are adapted from the GRAPPA and EULAR published recommendations and we acknowledge these source documents [4, 5]. Following development of this manuscript, source guideline developers were consulted for feedback.

#### Plan for review and update

The management of PsA is a rapidly evolving field with a number of new medications approved since the 2015 recommendations and further drugs currently in development. As EULAR and GRAPPA update their recommendations, these recommendations should also undergo periodic update.

## Discussion

The Institute of Medicine defines clinical guidelines as “systematically developed statements to assist practitioner and patient decisions about appropriate health care for specific clinical circumstances [9]. Hence, we aimed to adapt the most recently published GRAPPA and EULAR recommendations for the management of PsA for resource-poor settings. We followed the ADAPTE process and formulated PIPOH questions and conducted a systematic review of literature addressing these questions where it was not addressed by the source recommendations. However, evidence from the literature to answer the questions was weak or non-existent forcing us to resort to expert opinion. A research agenda in order to spur research into gaps in knowledge about management of PsA in resource-poor settings was formulated.

This process used published treatment recommendations for PsA, a heterogeneous disease affecting the skin and musculoskeletal structures, which have been developed and revised by GRAPPA [4] and EULAR [5], and most recently by the American College of Rheumatology [10]. However, these recommendations were developed based on data obtained largely in resource-replete settings and are more easily applicable to advanced economies. The applicability of these recommendations to resource-poor settings is questionable.

We chose to use the ADAPTE process and adapt existing recommendations rather than develop new recommendations. We believed that developing new recommendations from available literature would not be efficient since the current recommendations are based on review of recent developments in the field and are unlikely to be significantly different. We, therefore, chose to review the literature from the Americas and Africa to address questions relevant to management of PsA in resource-poor settings. Unfortunately, there is very little research done in resource-poor settings to address important practical questions about the management of PsA in the Americas and Africa.

We were able to engage rheumatologists and dermatologists as well as patients in developing these recommendations. The PIPOH questions were developed mainly by practitioners and patients from the Americas and Africa and their input was crucial in providing expert opinion for the adapted recommendations. The strong collaborative effort across continents sets the stage for designing studies to address unmet needs using the research network we have developed through this exercise.

The recommendations demonstrate that the goals of treatment, assessment of disease and associated comorbidities, and principles of safety and follow up are similar to the source recommendations. However, the type and severity of comorbidities are likely to be different in resource-poor settings. It is believed that the burden of concomitant infectious diseases such as TB, HB/CV, HIV, Chagas’ disease, and leishmaniasis is likely to be higher although high-quality studies showing high prevalence in PsA were lacking. Given the likelihood of adverse outcomes with newer immunomodulatory therapy, studies evaluating the safety and efficacy of these drugs in resource-poor settings are required but are currently lacking (and/or) are of poor quality. Pragmatic interventional and observational trials with the newer agents in resource-poor settings will benefit clinical decision-making and are on the research agenda.

Likewise, the prevalence of chronic disease is also rising in resource-poor settings [11]. Thus the management of PsA and associated non-communicable as well as infection-related comorbidities is challenging and will need to be addressed in future research studies given the lack of literature. We have provided expert opinion and acknowledge its inherent limitations.

One major unanswered question in the management of PsA is the safety and efficacy of combination therapy. Combination therapy (with multiple csDMARDs or a combination of csDMARDS and bDMARDs) is often used by clinicians in resource-replete as well as resource-poor settings but the evidence for efficacy and safety (especially with comorbidities) is lacking. Similarly, biosimilars and intended copies are increasingly available and being used with limited evidence about its safety and efficacy in patients with PsA. This is particularly relevant in the resource-poor setting where access to costly newer medications is poor and hence combination therapy or use of intended copies is likely to be more frequent in PsA resistant to monotherapy. Further research in this area is of utmost importance. One related clinical question is also how frequently to monitor patients. This is primarily a question of resources since the doctor-patient ratio and the resources for conducting laboratory tests are grossly inadequate in most countries. An efficient and cost-effective model is required but is yet to be developed. Moreover, educational programs to guide rheumatologists in the management of psoriasis when access to a dermatologist is poor, and to a dermatologist for the management of PsA when access to a rheumatologist or internist is difficult may improve care of PsA in these settings.

We did not include studies from the Asia-Pacific region in this exercise since APLAR was developing their own recommendations. However, we intend to collaborate with researchers and clinicians from that region to develop country or region-specific recommendations and share best practices. Moreover, we did not include the most recent American College of Rheumatology/National Psoriasis Foundation guidelines for the treatment of PsA [10] or the most recent update of the EULAR PsA treatment recommendations since these recommendations were published only after our literature review and guideline appraisals were completed.

Thus, the ILAR Treatment Recommendations for PsA were developed through the collaborative effort of researchers and clinicians from the Americas including Canada, Africa, and the UK. These recommendations intend to serve as reference for the management of PsA in resource-poor settings in the Americas and Africa. This paper illustrates the experience of an international working group in adapting existing recommendations to resource-poor setting. It highlights the need to conduct research on the management of PsA in these regions, sets a research agenda and intends to form the basis to conduct collaborative clinical research on the management of PsA in resource-poor settings.

## Electronic supplementary material

ESM 1 (DOCX 62 kb)
