# Supplementary material for: International league of associations for rheumatology recommendations for the management of psoriatic arthritis in resource-poor settings
Source: Clin Rheumatol. 2020 Jan 16;39(6):1839–50. doi: 10.1007/s10067-020-04934-7 (PMC7237392; doi:10.1007/s10067-020-04934-7)
Supplement: Supplementary file 1 — (DOCX 62 kb) [file 10067_2020_4934_MOESM1_ESM.docx]

**APPENDIX 1**

Search Completed By: Melanie Anderson

Information Specialist, University Health Network

Toronto, Canada.

**First Search strategy up to 2016**

|  | |  |  |  |
| --- | --- | --- | --- | --- |
| Database(s): Embase 1974 to 2016 November 23 |  | |  |  |
| Search Strategy: |  | |  |  |
| # | Searches | | Results | Annotations |
| 1 | psoriatic arthritis/ | | 16061 |  |
| 2 | (psoria* adj4 arthrit*).mp. | | 18447 |  |
| 3 | 1 or 2 | | 18447 |  |
| 4 | medically underserved/ or (underserv* or isolated or inaccessib*).tw. | | 1012254 |  |
| 5 | developing country/ or (developing or undevelop* or underdevelop* or "low income" or poverty or poor).tw. | | 1176656 |  |
| 6 | exp "south and central america"/ or ("south america*" or "central america*" or bra#il* or col#mbia* or argentin* or peru* or venezuela* or chile* or ecuador* or boliv* or paragua* or urugua* or guyan* or surinam* or guian* or falkland*).tw. | | 272948 |  |
| 7 | exp Africa/ or (africa* or niger* or ethiop* or egypt* or congo* or tanzan* or kenya* or sudan* or alger* or ugand* or morocc* or mozamb* or ghan* or angol* or ivory or ivoire or madagasc* or camero* or burkina or faso or mali* or malaw* or zambia* or senegal* or chad* or zimbabw* or rwand* or tunis* or somali* or guinea* or benin* or burundi* or togo* or eritrea* or sierra* or leone* or libya* or liberia* or mauritania* or namibia* or botswana* or gambia* or lesoth* or gabon* or mauriti* or swazi* or djibout* or comoro* or cape verd* or sahara* or mayott* or sao tome or seychell*).tw. | | 1217699 |  |
| 8 | indigenous health care/ or exp indigenous people/ or (indigenous or aborigin* or native?).tw. | | 243158 |  |
| 9 | exp african/ or exp central american/ or exp south american/ | | 29303 |  |
| 10 | or/4-9 | | 3603610 |  |
| 11 | 3 and 10 | | 1937 |  |
| 12 | exp Tuberculosis/ | | 200491 |  |
| 13 | tb.tw. | | 48249 |  |
| 14 | exp virus hepatitis/ | | 191632 |  |
| 15 | (hb or hc).tw. | | 78831 |  |
| 16 | hep b.tw. | | 328 |  |
| 17 | hep c.tw. | | 229 |  |
| 18 | exp Human immunodeficiency virus/ | | 238851 |  |
| 19 | exp Human immunodeficiency virus infection/ | | 340542 |  |
| 20 | exp tropical disease/ | | 149311 |  |
| 21 | exp parasitosis/ | | 327870 |  |
| 22 | exp virus infection/ | | 1011863 |  |
| 23 | exp bacterial infection/ | | 821606 |  |
| 24 | exp antiinfective agent/ | | 2742197 |  |
| 25 | hiv.tw. | | 322276 |  |
| 26 | (acquired adj4 immun*).tw. | | 30805 |  |
| 27 | malaria*.tw. | | 79091 |  |
| 28 | tst positive.tw. | | 393 |  |
| 29 | igra positive.tw. | | 111 |  |
| 30 | or/12-29 | | 4295351 |  |
| 31 | 11 and 30 | | 572 |  |
| 32 | limit 31 to embase | | 304 |  |

| Database(s): Ovid MEDLINE(R) 1946 to November Week 3 2016 |  |  |  |
| --- | --- | --- | --- |
| Search Strategy: |  |  |  |
| # | Searches | Results | Annotations |
| 1 | Arthritis, Psoriatic/ | 4838 |  |
| 2 | (psoria* adj4 arthrit*).mp. | 7931 |  |
| 3 | 1 or 2 | 7931 |  |
| 4 | Medically Underserved Area/ or (underserv* or isolated or inaccessib*).tw. | 853868 |  |
| 5 | Developing Countries/ or (developing or undevelop* or underdevelop* or "low income" or poverty or poor).tw. | 872281 |  |
| 6 | exp South America/ or ("south america*" or "central america*" or bra#il* or col#mbia* or argentin* or peru* or venezuela* or chile* or ecuador* or boliv* or paragua* or urugua* or guyan* or surinam* or guian* or falkland*).tw. | 198900 |  |
| 7 | exp Africa/ or (africa* or niger* or ethiop* or egypt* or congo* or tanzan* or kenya* or sudan* or alger* or ugand* or morocc* or mozamb* or ghan* or angol* or ivory or ivoire or madagasc* or camero* or burkina or faso or mali* or malaw* or zambia* or senegal* or chad* or zimbabw* or rwand* or tunis* or somali* or guinea* or benin* or burundi* or togo* or eritrea* or sierra* or leone* or libya* or liberia* or mauritania* or namibia* or botswana* or gambia* or lesoth* or gabon* or mauriti* or swazi* or djibout* or comoro* or cape verd* or sahara* or mayott* or sao tome or seychell*).tw. | 957454 |  |
| 8 | Health Services, Indigenous/ or (indigenous or aborigin* or native?).tw. | 210256 |  |
| 9 | african continental ancestry group/ or indians, central american/ or indians, south american/ | 43342 |  |
| 10 | or/4-9 | 2871249 |  |
| 11 | 3 and 10 | 659 |  |
| 12 | exp Tuberculosis/ | 189835 |  |
| 13 | tb.tw. | 33777 |  |
| 14 | Hepatitis C/ or Hepatitis B/ | 71684 |  |
| 15 | (hb or hc).tw. | 47944 |  |
| 16 | hep b.tw. | 85 |  |
| 17 | hep c.tw. | 38 |  |
| 18 | exp HIV/ | 105532 |  |
| 19 | exp HIV Infections/ | 283167 |  |
| 20 | Tropical Medicine/ | 6246 |  |
| 21 | exp Parasitic Diseases/ | 367152 |  |
| 22 | exp Virus Diseases/ | 888621 |  |
| 23 | exp "bacterial infections and mycoses"/ | 1378006 |  |
| 24 | exp Anti-Infective Agents/ | 1523064 |  |
| 25 | hiv.tw. | 281969 |  |
| 26 | (acquired adj4 immun*).tw. | 29222 |  |
| 27 | malaria*.tw. | 71280 |  |
| 28 | tst positive.tw. | 306 |  |
| 29 | igra positive.tw. | 53 |  |
| 30 | or/12-29 | 3492567 |  |
| 31 | 11 and 30 | 137 |  |

Database : LILACS

Search on : tuberculosis or tubercular or TB or "t.b." or hepatitis or hb or hc or hep b or hep c or hiv or parasite or parasites or parasitic or virus or viruses or viral or bacteria or bacterial or infection or infections or infecting or infects or infected or infections or infectious or infective or malaria or malarial or acquired immune or tst positive or igra postitive [Words] and psoriatic arthritis or psoriasis or PsA [Words]

References found : 159

**Second Search Strategy up to 2018**

**cMETHODS**

The search of the literature was conducted by a medical librarian (MA) using 5 databases: OVID Medline, OVID Embase, OVID Cochrane Central Register of Controlled Trials, LILACS and African Index Medicus(AIM). In each OVID database we used available subject headings for psoriatic arthritis, as well as underserved areas, developing countries, low income countries, and population groups who might be indexed instead of the geographic area.  Appropriate keywords for these concepts as well as the countries appearing in the World Bank Data Low and Middle Income lists* on February 5, 2018, were also used.  In LILACS and AIM searches involved keywords for psoriatic arthritis alone.  No date or language limits were applied.  Conference Abstracts were removed from the Embase results.  Complete strategies can be found in [[supplement? Appendix?]]

*  Found here: <https://data.worldbank.org/country>

**Complete Search Strategies**

Database(s): **Ovid MEDLINE(R) Epub Ahead of Print, In-Process & Other Non-Indexed Citations, Ovid MEDLINE(R) Daily and Ovid MEDLINE(R)**1946 to Present  
Search Strategy:

| **#** | **Searches** | **Results** |
| --- | --- | --- |
| 1 | Arthritis, Psoriatic/ | 5000 |
| 2 | (psoria* adj4 arthrit*).mp,kw. | 9435 |
| 3 | 1 or 2 | 9435 |
| 4 | Medically Underserved Area/ | 6473 |
| 5 | (underserv* or isolated or inaccessib*).mp,kw,oa,so,in,kw,cp,go,lg. | 902582 |
| 6 | Developing Countries/ | 69405 |
| 7 | ((developing or undevelop* or underdevelop* or "low income" or poverty or poor or "less developed" or underserved or under served or deprived or poor*) adj6 (countr* or nation* or population* or world? or econom*)).mp,kw,oa,so,in,kw,cp,go,lg. | 156702 |
| 8 | Health Services, Indigenous/ | 2708 |
| 9 | (indigenous or aborigin* or native?).mp,kw. | 222198 |
| 10 | african continental ancestry group/ or indians, central american/ or indians, south american/ | 39331 |
| 11 | (low* adj (gdp or gnp or gross domestic or gross national)).mp,kw,oa,so,in,kw,cp,go,lg. | 214 |
| 12 | (low adj3 middle adj3 countr*).mp,kw,oa,so,in,kw,cp,go,lg. | 10134 |
| 13 | transitional countr*.mp,kw,oa,so,in,kw,cp,go,lg. | 141 |
| 14 | (lmic or lmics or lami countr*).mp,kw,oa,so,in,kw,cp,go,lg. | 2471 |
| 15 | (Afghan* or Albania* or Algeria* or Angola* or Antigua* or Barbud* or Argentin* or Armenia* or Aruba* or Azerbaijan* or Bahrain* or Bangladesh* or Barbad* or Benin* or Byelarus* or Byelorus* or Belarus* or Belorus* or Beliz* or Bhutan* or Bolivia* or Bosnia* or Herzegovin* or Hercegovin* or Botswan* or Brasil* or Brazil* or Bulgaria* or Burkina Faso* or Burkina Fasso* or Upper Volta* or Burundi* or Urundi* or Cambodia* or Khmer Republic or Kampuchea* or Cameroon* or Cameron* or Cape Verde* or Central African Republic or Chad* or Chile* or China or chinese or Colombia* or Comoros* or Comoro Islands or Comores or Mayott* or Congo* or Zair* or Costa Rica* or Cote d'Ivoire or Ivory Coast or Croatia* or Cuba* or Cyprus or cyprian or Czechoslovakia* or Czech Republic or Slovakia* or Slovak Republic or Djibouti* or French Somaliland or Dominica* or East Timor or East Timur or Timor Leste or Ecuador* or Egypt* or United Arab Republic or El Salvador* or Eritrea* or Estonia* or Ethiopia* or Fiji* or Gabon* or Gambia* or Gaza* or Georgia Republic or Georgian Republic or georgian or Ghana* or Gold Coast or Greece or greek or Grenada* or Guatemala* or Guinea* or Guam* or Guiana* or Guyana* or Haiti* or Hondura* or Hungar* or India* or Maldiv* or Indonesia* or Iran* or Iraq* or Isle of Man or Jamaica* or Jordan* or Kazakh* or Kenya* or Kiribati* or Korea* or Kosovo* or Kyrgyz* or Kirghiz* or Kirgiz* or Lao PDR or Laos* or Latvia* or Leban* or Lesotho* or Basutoland or Liberia* or Libya* or Lithuania* or Macedonia* or Madagascar* or Malagasy Republic or Malay* or Sabah* or Sarawak* or Malawi* or Nyasaland* or Mali* or Malta* or Marshall Island* or Maurit* or Agalega Island* or Mexic* or Micronesia* or Middle East* or Moldova* or Moldovia* or Mongolia* or Montenegr* or Morocc* or Ifni* or Mozambiq* or Myanmar* or Myanma or Burma* or Namibia* or Nepal* or Netherlands Antill* or New Caledonia* or Nicaragua* or Niger* or Northern Mariana Island* or Oman* or Muscat* or Pakistan* or Palau* or Palestin* or Panama* or Paragua* or Peru* or Phi?lippin* or Poland or polish or Portug* or Puerto Ric* or Romania* or Rumania* or Roumania* or Russia* or Rwanda* or Ruanda* or Saint Kitts* or St Kitts or Nevis* or Saint Lucia* or St Lucia* or Saint Vincent* or St Vincent* or Grenadin* or Samoa* or Navigator Island* or Sao Tome* or Saudi Arabia* or saudi or Senegal* or Serbia* or Montenegr* or Seychelles or Sierra Leone or Slovenia* or Sri Lanka* or Ceylon* or Solomon Islands or Somalia* or South Africa* or Sudan* or Surinam* or Swaziland or swazi or Syria* or Tajik* or Tadjik* or Tadzhik* or Tanzania* or Thailand or thai or Togo or Togolese Republic or Tonga* or Trinidad* or Tobag* or Tunisia* or Turkey or turkish or Turkmenistan* or Turkmen* or Uganda* or Ukrain* or Urugua* or USSR or Soviet Union or Union of Soviet Socialist Republics or Uzbek* or Vanuat* or New Hebrides or Venezuela* or Vietnam* or Viet Nam* or West Bank or Yemen* or Yugoslavia* or Zambia* or Zimbabw* or Rhodesia* or cabo verd* or tuvalu or gaza).mp,kw,oa,so,in,kw,cp,go,lg. | 7042034 |
| 16 | or/4-15 | 7861871 |
| 17 | 3 and 16 | 2088 |

Database(s): **Embase**1974 to 2018 February 21  
Search Strategy:

| **#** | **Searches** | **Results** |
| --- | --- | --- |
| 1 | psoriatic arthritis/ | 17257 |
| 2 | (psoria* adj4 arthrit*).mp. | 20468 |
| 3 | 1 or 2 | 20468 |
| 4 | (underserv* or isolated or inaccessib*).mp,gc,in,jn,tj. | 1075272 |
| 5 | developing country/ | 88593 |
| 6 | ((developing or undevelop* or underdevelop* or "low income" or poverty or poor or "less developed" or underserved or under served or deprived or poor*) adj6 (countr* or nation* or population* or world? or econom*)).mp,gc,in,jn,tj. | 181483 |
| 7 | indigenous health care/ | 474 |
| 8 | (indigenous or aborigin* or native?).mp. | 255279 |
| 9 | black person/ or african brazilian/ or african caribbean/ or exp hispanic/ or ancestry group/ or asian continental ancestry group/ or exp indigenous people/ | 84385 |
| 10 | (low* adj (gdp or gnp or gross domestic or gross national)).mp,gc,in,jn,tj. | 307 |
| 11 | (low adj3 middle adj3 countr*).mp,gc,in,jn,tj. | 11546 |
| 12 | transitional countr*.mp,gc,in,jn,tj. | 202 |
| 13 | (lmic or lmics or lami countr*).mp,gc,in,jn,tj. | 3018 |
| 14 | (Afghan* or Albania* or Algeria* or Angola* or Antigua* or Barbud* or Argentin* or Armenia* or Aruba* or Azerbaijan* or Bahrain* or Bangladesh* or Barbad* or Benin* or Byelarus* or Byelorus* or Belarus* or Belorus* or Beliz* or Bhutan* or Bolivia* or Bosnia* or Herzegovin* or Hercegovin* or Botswan* or Brasil* or Brazil* or Bulgaria* or Burkina Faso* or Burkina Fasso* or Upper Volta* or Burundi* or Urundi* or Cambodia* or Khmer Republic or Kampuchea* or Cameroon* or Cameron* or Cape Verde* or Central African Republic or Chad* or Chile* or China or chinese or Colombia* or Comoros* or Comoro Islands or Comores or Mayott* or Congo* or Zair* or Costa Rica* or Cote d'Ivoire or Ivory Coast or Croatia* or Cuba* or Cyprus or cyprian or Czechoslovakia* or Czech Republic or Slovakia* or Slovak Republic or Djibouti* or French Somaliland or Dominica* or East Timor or East Timur or Timor Leste or Ecuador* or Egypt* or United Arab Republic or El Salvador* or Eritrea* or Estonia* or Ethiopia* or Fiji* or Gabon* or Gambia* or Gaza* or Georgia Republic or Georgian Republic or georgian or Ghana* or Gold Coast or Greece or greek or Grenada* or Guatemala* or Guinea* or Guam* or Guiana* or Guyana* or Haiti* or Hondura* or Hungar* or India* or Maldiv* or Indonesia* or Iran* or Iraq* or Isle of Man or Jamaica* or Jordan* or Kazakh* or Kenya* or Kiribati* or Korea* or Kosovo* or Kyrgyz* or Kirghiz* or Kirgiz* or Lao PDR or Laos* or Latvia* or Leban* or Lesotho* or Basutoland or Liberia* or Libya* or Lithuania* or Macedonia* or Madagascar* or Malagasy Republic or Malay* or Sabah* or Sarawak* or Malawi* or Nyasaland* or Mali* or Malta* or Marshall Island* or Maurit* or Agalega Island* or Mexic* or Micronesia* or Middle East* or Moldova* or Moldovia* or Mongolia* or Montenegr* or Morocc* or Ifni* or Mozambiq* or Myanmar* or Myanma or Burma* or Namibia* or Nepal* or Netherlands Antill* or New Caledonia* or Nicaragua* or Niger* or Northern Mariana Island* or Oman* or Muscat* or Pakistan* or Palau* or Palestin* or Panama* or Paragua* or Peru* or Phi?lippin* or Poland or polish or Portug* or Puerto Ric* or Romania* or Rumania* or Roumania* or Russia* or Rwanda* or Ruanda* or Saint Kitts* or St Kitts or Nevis* or Saint Lucia* or St Lucia* or Saint Vincent* or St Vincent* or Grenadin* or Samoa* or Navigator Island* or Sao Tome* or Saudi Arabia* or saudi or Senegal* or Serbia* or Montenegr* or Seychelles or Sierra Leone or Slovenia* or Sri Lanka* or Ceylon* or Solomon Islands or Somalia* or South Africa* or Sudan* or Surinam* or Swaziland or swazi or Syria* or Tajik* or Tadjik* or Tadzhik* or Tanzania* or Thailand or thai or Togo or Togolese Republic or Tonga* or Trinidad* or Tobag* or Tunisia* or Turkey or turkish or Turkmenistan* or Turkmen* or Uganda* or Ukrain* or Urugua* or USSR or Soviet Union or Union of Soviet Socialist Republics or Uzbek* or Vanuat* or New Hebrides or Venezuela* or Vietnam* or Viet Nam* or West Bank or Yemen* or Yugoslavia* or Zambia* or Zimbabw* or Rhodesia* or cabo verd* or tuvalu or gaza).mp,gc,in,jn,tj. | 8132546 |
| 15 | or/4-14 | 9081925 |
| 16 | 3 and 15 | 5604 |
| 17 | limit 16 to conference abstract status | 1990 |
| 18 | 16 not 17 | 3614 |

Database(s): **EBM Reviews - Cochrane Central Register of Controlled Trials**January 2018  
Search Strategy:

| **#** | **Searches** | **Results** |
| --- | --- | --- |
| 1 | psoriatic arthritis/ or Arthritis, Psoriatic/ | 207 |
| 2 | (psoria* adj4 arthrit*).mp. | 987 |
| 3 | 1 or 2 | 987 |
| 4 | (underserv* or isolated or inaccessib*).mp,in,jn,jw,so. | 9997 |
| 5 | developing country/ or Medically Underserved Area/ or Developing Countries/ | 800 |
| 6 | ((developing or undevelop* or underdevelop* or "low income" or poverty or poor or "less developed" or underserved or under served or deprived or poor*) adj6 (countr* or nation* or population* or world? or econom*)).mp,in,jn,jw,so. | 5147 |
| 7 | indigenous health care/ or Health Services, Indigenous/ | 28 |
| 8 | (indigenous or aborigin* or native?).mp. | 2755 |
| 9 | black person/ or african brazilian/ or african caribbean/ or exp Hispanic Americans/ or ancestry group/ or asian continental ancestry group/ or exp indigenous people/ or african continental ancestry group/ or indians, central american/ or indians, south american/ | 3735 |
| 10 | (low* adj (gdp or gnp or gross domestic or gross national)).mp,in,jn,jw,so. | 39 |
| 11 | (low adj3 middle adj3 countr*).mp,in,jn,jw,so. | 529 |
| 12 | transitional countr*.mp,in,jn,jw,so. | 3 |
| 13 | (lmic or lmics or lami countr*).mp,in,jn,jw,so. | 120 |
| 14 | (Afghan* or Albania* or Algeria* or Angola* or Antigua* or Barbud* or Argentin* or Armenia* or Aruba* or Azerbaijan* or Bahrain* or Bangladesh* or Barbad* or Benin* or Byelarus* or Byelorus* or Belarus* or Belorus* or Beliz* or Bhutan* or Bolivia* or Bosnia* or Herzegovin* or Hercegovin* or Botswan* or Brasil* or Brazil* or Bulgaria* or Burkina Faso* or Burkina Fasso* or Upper Volta* or Burundi* or Urundi* or Cambodia* or Khmer Republic or Kampuchea* or Cameroon* or Cameron* or Cape Verde* or Central African Republic or Chad* or Chile* or China or chinese or Colombia* or Comoros* or Comoro Islands or Comores or Mayott* or Congo* or Zair* or Costa Rica* or Cote d'Ivoire or Ivory Coast or Croatia* or Cuba* or Cyprus or cyprian or Czechoslovakia* or Czech Republic or Slovakia* or Slovak Republic or Djibouti* or French Somaliland or Dominica* or East Timor or East Timur or Timor Leste or Ecuador* or Egypt* or United Arab Republic or El Salvador* or Eritrea* or Estonia* or Ethiopia* or Fiji* or Gabon* or Gambia* or Gaza* or Georgia Republic or Georgian Republic or georgian or Ghana* or Gold Coast or Greece or greek or Grenada* or Guatemala* or Guinea* or Guam* or Guiana* or Guyana* or Haiti* or Hondura* or Hungar* or India* or Maldiv* or Indonesia* or Iran* or Iraq* or Isle of Man or Jamaica* or Jordan* or Kazakh* or Kenya* or Kiribati* or Korea* or Kosovo* or Kyrgyz* or Kirghiz* or Kirgiz* or Lao PDR or Laos* or Latvia* or Leban* or Lesotho* or Basutoland or Liberia* or Libya* or Lithuania* or Macedonia* or Madagascar* or Malagasy Republic or Malay* or Sabah* or Sarawak* or Malawi* or Nyasaland* or Mali* or Malta* or Marshall Island* or Maurit* or Agalega Island* or Mexic* or Micronesia* or Middle East* or Moldova* or Moldovia* or Mongolia* or Montenegr* or Morocc* or Ifni* or Mozambiq* or Myanmar* or Myanma or Burma* or Namibia* or Nepal* or Netherlands Antill* or New Caledonia* or Nicaragua* or Niger* or Northern Mariana Island* or Oman* or Muscat* or Pakistan* or Palau* or Palestin* or Panama* or Paragua* or Peru* or Phi?lippin* or Poland or polish or Portug* or Puerto Ric* or Romania* or Rumania* or Roumania* or Russia* or Rwanda* or Ruanda* or Saint Kitts* or St Kitts or Nevis* or Saint Lucia* or St Lucia* or Saint Vincent* or St Vincent* or Grenadin* or Samoa* or Navigator Island* or Sao Tome* or Saudi Arabia* or saudi or Senegal* or Serbia* or Montenegr* or Seychelles or Sierra Leone or Slovenia* or Sri Lanka* or Ceylon* or Solomon Islands or Somalia* or South Africa* or Sudan* or Surinam* or Swaziland or swazi or Syria* or Tajik* or Tadjik* or Tadzhik* or Tanzania* or Thailand or thai or Togo or Togolese Republic or Tonga* or Trinidad* or Tobag* or Tunisia* or Turkey or turkish or Turkmenistan* or Turkmen* or Uganda* or Ukrain* or Urugua* or USSR or Soviet Union or Union of Soviet Socialist Republics or Uzbek* or Vanuat* or New Hebrides or Venezuela* or Vietnam* or Viet Nam* or West Bank or Yemen* or Yugoslavia* or Zambia* or Zimbabw* or Rhodesia* or cabo verd* or tuvalu or gaza).mp,in,jn,jw,so. | 200003 |
| 15 | or/4-14 | 213285 |
| 16 | 3 and 15 | 157 |

February 22, 2018

| Database : | **LILACS** |
| --- | --- |
| Search on : | **psoriatic arthritis or psoriasis or PsA [Words]** |
| References found : | **1604** [[refine](http://bases.bireme.br/cgi-bin/wxislind.exe/iah/online/)] |

February 22, 2018

African Index Medicus

**38 electronic documents or title(s) found 'psoriatic arthritis'**

In Titles, Abstract and notes, descriptors, keywords

**APPENDIX 2**

| Tool 15 Modified.  EULAR Principle B taken as an example | | | | | |
| --- | --- | --- | --- | --- | --- |
| **EULAR Principle** | | | | | |
| \| B. Treatment of patients with PsA should aim at the best care and must be based on a shared decision between the patient and the rheumatologist, considering efficacy, safety and costs. \|  \|  \| \| --- \| --- \| --- \| | | | | | |
|  |  |  | **Accept as is** | ***Modify** | **Reject** |
| **Overall, the principle is acceptable** | | |  |  |  |
| The population matches with the one described for this project | | |  |  |  |
| Its implementation will benefit the population | | |  |  |  |
| It is compatible with the culture and values of the population | | |  |  |  |
|  |  |  | **Accept as is** | ***Modify** | **Reject** |
| **Overall, the principle is applicable** | | |  |  |  |
| The intervention is applicable to the patients in this context | | |  |  |  |
| The intervention/equipment is available in this context | | |  |  |  |
| The expertise is available in this context | | |  |  |  |
| There are no constraints, legislations, policies, or resources that would impede its implementation | | |  |  |  |
| ***Please state your desired modification to this principle** | | |  | | |
|  |  | **Comments** |  | | |

**ADAPTATION PHASE Decision and Selection Module**

**Tool 15 Modified:  Evaluation sheet - Acceptability/Applicability**

Decision and selection made on the content of the source guidelines

Definitions:

**Acceptable:**It should put it into practice

**Applicable:**  Physicians are able to put it into practice
